# Drawing Inspiration from Human Brain Networks: Construction of Interconnected Virtual Networks

**DOI:** 10.3390/s18041133

**Published:** 2018-04-08

**Authors:** Masaya Murakami, Daichi Kominami, Kenji Leibnitz, Masayuki Murata

**Affiliations:** 1 Graduate School of Information Science and Technology, Osaka University, 1-5, Yamadaoka, Suita 565-0871, Osaka, Japan; murata@ist.osaka-u.ac.jp; 2 Graduate School of Economics, Osaka University, 1-7, Machikaneyama-cho, Toyonaka 560-0043, Osaka, Japan; d-kominami@econ.osaka-u.ac.jp; 3 Center for Information and Neural Networks (CiNet), 1-4, Yamadaoka, Suita 565-0871, Osaka, Japan; leibnitz@nict.go.jp

**Keywords:** Internet of Things, brain networks, virtual networks, wireless sensor networks

## Abstract

Virtualization of wireless sensor networks (WSN) is widely considered as a foundational block of edge/fog computing, which is a key technology that can help realize next-generation Internet of things (IoT) networks. In such scenarios, multiple IoT devices and service modules will be virtually deployed and interconnected over the Internet. Moreover, application services are expected to be more sophisticated and complex, thereby increasing the number of modifications required for the construction of network topologies. Therefore, it is imperative to establish a method for constructing a virtualized WSN (VWSN) topology that achieves low latency on information transmission and high resilience against network failures, while keeping the topological construction cost low. In this study, we draw inspiration from inter-modular connectivity in human brain networks, which achieves high performance when dealing with large-scale networks composed of a large number of modules (i.e., regions) and nodes (i.e., neurons). We propose a method for assigning inter-modular links based on a connectivity model observed in the cerebral cortex of the brain, known as the exponential distance rule (EDR) model. We then choose endpoint nodes of these links by controlling inter-modular assortativity, which characterizes the topological connectivity of brain networks. We test our proposed methods using simulation experiments. The results show that the proposed method based on the EDR model can construct a VWSN topology with an optimal combination of communication efficiency, robustness, and construction cost. Regarding the selection of endpoint nodes for the inter-modular links, the results also show that high assortativity enhances the robustness and communication efficiency because of the existence of inter-modular links of two high-degree nodes.

## 1. Introduction

Wireless sensor network (WSN) [1] refers to an ad hoc type of network that comprises spatially-distributed autonomous sensor devices. These sensor devices are connected to each other via a network, and they monitor the environment of a target area. Recent advancements in information and communication technology (ICT) has led to miniaturized and sophisticated sensor devices (e.g., low-power wide area (LPWA) networks [2,3]), and WSNs are the foundational blocks for further advanced network systems, such as edge computing [4,5] or fog computing [6,7], which attract a great deal of attention for realizing the Internet of things (IoT) and cyber-physical systems (CPS) [8,9,10,11]. In these scenarios, a number of ICT application services and other social infrastructure will be integrated and implemented on the Internet.

**Figure 1:**
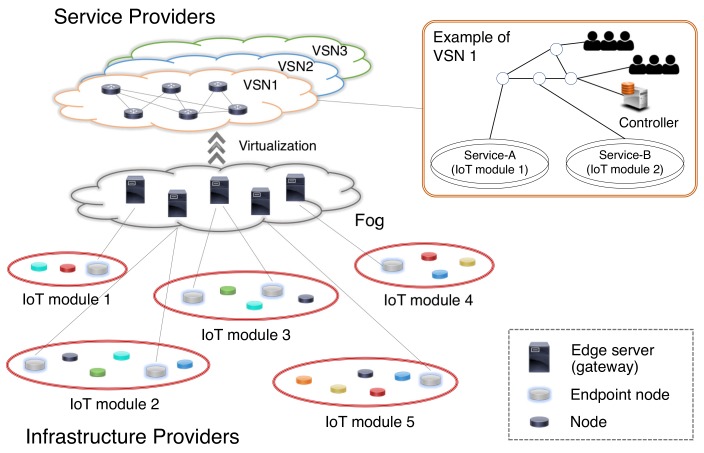
Architecture for virtualized wireless sensor networks (VWSNs).

Virtualization techniques, such as network function virtualization (NFV) [12,13], software defined networks (SDN) [14,15], and network slicing [16,17], have also been studied in combination with WSNs [18,19,20,21,22], and are considered key technologies to construct network systems for edge computing [23,24,25,26] and IoT [27,28,29,30]. A virtualized WSN (VWSN) is composed of two network layers: the infrastructure layer and the service layer (Figure 1). Multiple providers deploy physical network resources that can analyze the environment, collect and propagate data, and form IoT modules. Therefore, an infrastructure layer that comprises an interconnected structure of multiple IoT modules is realized. The service layer is virtually constructed by combining multiple IoT modules on this infrastructure layer, wirelessly connecting the edge servers, which process/transmit data and behave as gateways, based on millimeter-wave beam-forming techniques [31,32]. In this scenario, the nodes in Figure 1 correspond to various types of IoT devices; some of them operate as endpoint nodes, which have the ability to serve as a representative nodes to communicate with the edge servers. The administrator of a virtualized service networks (VSN) operates edge servers and virtualized wireless connections among the IoT modules.

This virtualization architecture has several advantages for future IoT scenarios [18,19,20,22]. For instance, even if the type of services to be implemented on the VWSN is not envisaged beforehand, administrators can construct the service layer for any purpose while flexibly reusing physical resources at relatively low costs. Second, virtualization in WSN can address the heterogeneity in the infrastructure layer, wherein diverse types of physical network modules are individually deployed by the infrastructure providers. In addition, separating infrastructure and service layers results in simplified operation and accelerated development.

VWSN architecture provides features that are conducive to the realization of IoT technology; however, no strategies have been proposed that efficiently generate virtualized topologies, wherein numerous networks are mutually interconnected. Virtualization techniques for the WSNs enable the administrators of the service providers to flexibly construct the VSN topologies [18,19,21,22]. At the same time, the number of ICT services provided over the Internet has been skyrocketing, and their contents become more and more sophisticated [8,9,10,11]. These situations demand frequent modification of the VWSN topologies: the administrators require addition/removal of network elements (i.e., nodes and links) on the existing VSNs, and the new VSNs will be constructed every time a new service is launched. Furthermore, in the future, the Internet will be comprised of millions of interconnected IoT devices, and a substantial computational cost will be incurred when calculating the optimal topologies for the VSNs. Therefore, although optimizing topology shapes is virtually impossible due to the enormous number of devices and services, it is imperative to discover appropriate ways for constructing VWSN topologies that achieve a high level of performance, quick communication between any pair of nodes, high resilience against failures of network components, and low cost to sustain the wireless connectivity, prior to the determination of the optimal shape of topologies.

In recent years, the application of human brain networks in the engineering field has attracted significant research attention because brain networks possess excellent characteristics that have been optimized through the process of evolution [33,34]. However, studies that have discussed the application of brain networks to information networking systems, particularly for the aforementioned modularly interconnected networks, are limited.

Remarkable advances of graph-theoretic analysis in neuroscience (e.g., functional Magnetic Resonance Imaging (fMRI)) have revealed that the modular structure of brain networks enables them to contain tens of billions of neurons, which can help a brain network adapt itself for a wide variety of tasks [33]. The inter-modular connectivity also enables the highly reliable information processing system of the brain, while optimizing the tradeoff between performance and metabolism [35]. Therefore, brain networks are considered to have fundamental characteristics that are necessary for designing an interconnected network of VWSNs. In this study, we investigate the relationship between the performance of information networks and the shape of the network topology based on recent findings in the field of neuroscience. In particular, we attempt to answer the following questions: *“which pair of modules should be connected by inter-modular links?”* and *“which nodes within modules should be selected as endpoints of these inter-modular links?”*.

First, we focus on the exponential distance rule (EDR) [35], which is a network model based on the modular connectivity in the cerebral cortex of a mammalian brain. The cerebral cortex is divided into multiple regions based on the local functional roles of those regions. The EDR model can reproduce the connectivity structure among those regions in the brain—the neural links among the areas are created based on a probability function that exponentially decays with inter-areal distance. In [35], Ercsey et al. showed that network topologies generated using the EDR model are similar to the topologies obtained from the fMRI experiments in terms of graph-topological features. The EDR model also explains how the human brain optimizes performance by considering the metabolic cost and inter-modular link length. In our previous work [36], we proposed a method to construct a VWSN topology and evaluated the performances regarding communication efficiency and wiring costs. In this study, we further reveal the performance of robustness against network failure, in relation to the communication efficiency and wiring costs.

However, the EDR model does not consider the endpoints of inter-modular links; therefore, this study accounts for the assortativity, which is defined as the correlation of node degrees in networks. Node degree is among the simplest and the most common centrality measures, and is used for measuring nodal importance on a network topology. In a network with high assortativity, a pair of nodes is likely to be interconnected if the two nodes have a similar node degree. By contrast, in networks with low assortativity, any two nodes are connected if they have a dissimilar node degree [37]. As for the brain network, which is composed of multiple modules, assortativity can indicate different behaviors depending on whether the focus is on the connections between the modules or within the modules as well as depending on whether the connections between the modules are strong or weak. High reliability of brain networks can be attributed to the topological connectivity, which is based on the assortativity between and within network modules [38,39].

The objective of this study is to design inter-modular network topologies for a VWSN that are robust against environmental changes and provide high communication efficiency at relatively low wiring costs. For the construction of the objective topology, we first assign links between modules based on our proposed method [36]. Second, by taking assortativity into account, we propose a method to assign inter-modular links on nodes within modules. The effects of the EDR model and assortativity can be controlled by a single parameter for each. Evaluation results reveal that the configuration of these two parameters is of critical importance to the performance: small communication delay between nodes, high robustness against network component failures, and low cost for constructing a VWSN topology. Therefore, the results of this study will guide future studies on the construction of inter-modular network topologies for VWSN that can realize high performance at relatively low wiring costs.

This paper is organized as follows. In Section 2, we present some related work about brain networks. Section 3 describes the method for constructing a VWSN topology that draws inspiration from brain networks. Then, Section 4 provides the evaluation results of our proposed method. Finally, Section 5 presents our conclusions.

## 2. Related Work

### 2.1. Modular Human Brain Networks

The human brain can be regarded as a complex network comprising neuronal cell bodies that reside in the cortical gray matter regions joined by myelin-insulated axons. Recent advancements in neuroimaging techniques have enabled the analysis of the human brain at higher spatial resolutions. Previous studies have examined the structural network of the brain as represented by anatomical connections among the regions of interest [40,41,42].

To the best of the authors’ knowledge, small-world and scale-free properties have been studied as main characteristics of brain networks [40,41,42,43]. Therefore, existing studies have not focused on other topological properties of brain networks such as the hierarchical modular structure. Hierarchical modularity is considered to be associated with sparseness, robustness, transmission of signals, maintenance of dynamic activity, and adaptive evolution [34,44,45]. In other words, the modular structure is closely associated with the performance of the human brain, including robustness, communication efficiency, scalability, and metabolic cost. In this study, we focus on the similarity between the brain networks and information networks, and apply structural properties of brain networks to construct VWSN topologies with high performance.

#### 2.1.1. Cerebral Cortical Inter-Modular Connectivity Model

Brain networks have recently been investigated from a topological viewpoint of complex networks. Moreover, previous studies have revealed the structure of brain networks in terms of small-world or scale-free properties. Although network models based on these two properties can generate topologies with characteristics found in brain networks (e.g., high modularity and low hop count), they do not consider the geometrical constraints [46]. Regarding the connectivity in brain networks, geometrical constraints should be considered because the metabolic cost of connecting two neurons increases with increasing length of the axons (i.e., physical distance between the neurons). Connecting distant neurons accelerates the information integration process in brain networks. However, long connections can add to the metabolic costs. In other words, we can conjecture that the connectivity structure of brain networks, i.e., the trade-off between metabolic cost and communication efficiency, has been optimized through the evolutionary process [47]. Regarding information networks, reducing the costs incurred owing to communication distances is necessary for both the wired and wireless networks. Long communication distances require higher wiring costs for the laying of physical cables for wired networks, and higher transmission power to overcome signal attenuation and interference in the case of wireless networks. At the same time, long connections are necessary for quick information transmission and robust connectivity.

Ercsey et al. proposed a novel network model [35] called the EDR model that is based on the neural connectivity in the cerebral cortex of the macaque monkey. When analyzing the neural connectivity, the entire macaque cortex was divided into 91 regions of interest, i.e., areas of neurons with similar functions. The nodes in the resulting network topology represent each cortical area. In the process, 29 of the spatially distributed 91 cortical areas are selected such that the subgraph of the 29 areas can completely estimate the connectivity for the entire network. Retrograde tracer injections into those 29 areas revealed that 6,494,974 neural links and 1615 inter-areal links were present. The analysis revealed that the existence probability p(d) of inter-areal connections exponentially decays with the inter-areal distance, which can be represented as follows:

(1) p(d)=cexp(-λd),

where *c* denotes the normalization constant, *d* is the inter-areal distance, and λ is a parameter. For the approximation of the cerebral connectivity, $\lambda =0.180\;{\mathrm{mm}}^{-1}$ is used in the EDR model [35]. It should be noted that links in the resulting topology are assigned weights corresponding to multiple neural connections between the areas. Given the trade-off between metabolic cost and performance, pairs of neurons that are in close proximity tend to have a higher number of connections; in addition, a few long-distance connections are present to accelerate information integration. Even though the EDR model is a relatively simple model that is controlled by only a single parameter λ, it can sufficiently reproduce various properties of topological connectivity in the cerebral network, such as communication efficiency, distribution of cliques, eigenvector spectra, and presence of a core structure [35].

#### 2.1.2. Assortativity in Human Brain Networks

Assortativity, which indicates the correlation of node degrees, is a common characteristic that is used for the evaluation of complex networks. High assortativity implies that nodes are preferentially connected if their degrees are similar. By contrast, in the case of low assortativity, nodes of different degree are likely to be connected. Newman [37] proposed the concept of *global assortativity* to measure the assortativity of an entire network. In addition, *universal assortativity* was introduced to evaluate the assortativity of any part of a network [48]. This universal assortativity was also used to define the assortativity between networks in our previous work [39].

In [37], Newman proposed a method to measure the assortativity of a network topology by using the *global assortativity coefficient*. The global assortativity coefficient is calculated based on the remaining degree distribution q(k), as follows:

(2) $$q\left(k\right)=\frac{\left(k+1)p(k+1\right)}{\sum_{j}jp\left(j\right)},$$

where p(k) is called degree distribution, which denotes the probability that a randomly selected node has node degree *k*; q(k) is referred to as the remaining degree distribution, which denotes the probability that either endpoint nodes of a randomly selected link have the remaining degree *k*. Here, the remaining degree of a node refers to the ordinary node degree minus the node itself. The global assortativity coefficient *r* is defined as follows:

(3) $$r=\frac{1}{\sigma_{q}^{2}}(E\left[\left(J-U_{q}\right)\left(K-U_{q}\right)\right]),$$

where *J* and *K* denote variables of the remaining degree; both have the same expected value $U_{q}=\sum_{j}jq\left(j\right)$. The term $\sigma_{q}^{2}=\sum_{l}j^{2}q\left(j\right)-(\sum_{k}kq\left(k\right){)}^{2}$ denotes the variance of the remaining degree distribution q(k). The positive and negative values of *r* imply that a network is assortative and disassortative, respectively. When *r* tends to zero, the network becomes non-assortative; the shape of a network becomes similar to that of a random network. Theoretically, the range of feasible values of *r* is [-1,1]; however, its range is rendered smaller due to the degree distribution.

Then, the *universal assortativity coefficient* $\rho_{l}$ on a link *l* can be introduced, given q(k). This coefficient corresponds to the contribution of an individual link to the global assortativity coefficient *r*. Therefore, $\rho_{l}$ is defined as follows:

(4) $$\rho_{l}=\frac{\left(j-U_{q}\right)\left(k-U_{q}\right)}{M\sigma_{q}^{2}},$$

where *j* and *k* denote the remaining degrees of the two endpoints of link *l*. Here, *M* denotes the total number of links in the network. Then, the universal assortativity coefficient ρ can be defined as follows:

(5) $$\rho =\sum_{l\in S}\rho_{l}=\sum_{l\in S}\frac{\left(J-U_{q}\right)\left(K-U_{q}\right)}{M\sigma_{q}^{2}},$$

where *S* denotes a set of links between modules. The assortativity between networks is determined using Equation (5). It can be said that the universal assortativity ρ is a part of global assortativity *r*. Hence, ρ is equal to *r* when *S* corresponds to all the nodes between modules. When $\rho_{l}>0$, the link is called an assortative link; when $\rho_{l}<0$, the link is called a disassortative link. A link with $\rho_{l}=0$ has no correlation.

With respect to human brain networks, it has been shown that the connectivity between modules exhibits assortative mixing when both strong and weak connections are considered [39]. Therefore, it can be inferred that the assortative connections facilitate communication between modules in the human brain. In contrast, when only strong links were considered, inter-modular connectivity showed disassortative mixing, which can accelerate concurrent and robust processing between two modules.

## 3. Method to Construct a VWSN Network Topology

**Figure 2:**
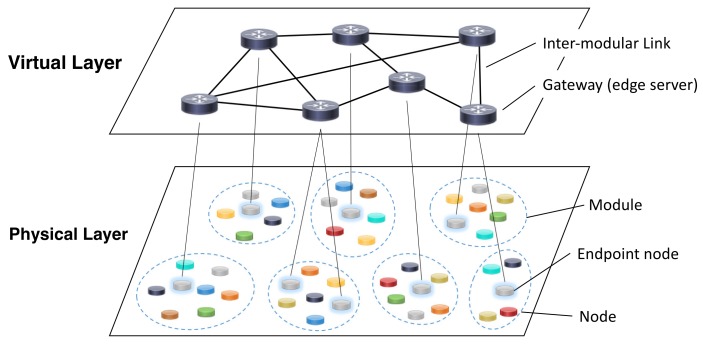
Architecture for virtualized wireless sensor network.

Virtualization in WSN is expected to play an important role on the IoT scenario [18,19,20,21,22], as explained in Section 2.1.2. It has enabled the construction of a VWSN topology over distributed wireless network resources with high flexibility and efficiency. In this study, we assume that a VWSN network is composed of two layers: *physical layer* and *virtual layer* (see Figure 2). On the physical layer, the physical network resources are deployed and connect to each other, and form heterogeneous network modules. Subsequently, the modules on the physical layer virtually connect to each other via wireless connections of inter-modular links between gateways (edge servers) and form the virtual layer. Regarding the assignment of endpoints of inter-modular links, all the nodes on the physical layer cannot behave as endpoints in a practical sense because the performance or role of the devices differ from each other. However, in this evaluation, we assume that all nodes have the ability to serve as an endpoint node. Therefore, our objective is to reveal the type of nodes that should be represented as an endpoint node from a topological viewpoint.

In the following subsections, the proposed method for constructing the topology of the virtual layer for the VWSN network architecture is described. First, the EDR model is modified and utilized to control the deployment of inter-modular links. Then, assortativity is applied to determine the assignment of endpoints of inter-modular links on nodes in the modules.

### 3.1. Physical Layer Assumption

Let us assume a simple network as the physical layer, which consists of multiple WSN modules. First, the given square area with a side length of *E* is divided into *M* smaller evenly sized squares. Each area has the same number of $N^{\prime }=N/M$ nodes, and, in total, *N* nodes are deployed on the entire square region. Although there are many other possible ways to deploy modules, the aforementioned deployment sufficiently achieves our objective, which aims to reveal the relation between the performances and the geometrical distance.

The node degree distribution in each module is configured such that it follows a Gaussian or power-law distribution, which are commonly observed in topologies of complex networks [49]. $L_{intra}$ links are deployed inside each module to form a connected topology. An intra-modular link does not have a direction or weight. Here, we assume that a gateway node is located at the geometrical center of each module.

### 3.2. Virtual Layer Construction

In this model, the distance between any two modules is calculated based on Euclidean distance between the coordinates of the gateways. We assume that modules are wirelessly interconnected through these gateway nodes according to the probability function in the EDR model, thus forming inter-modular links. In this manner, the virtual layer is constructed. A certain number of $L_{inter}$ inter-modular links are deployed on each network. Each inter-modular link does not have a direction or weight, but a pair of modules can have more than one inter-modular link.

For creating inter-modular links, we redefine Equation (1) as Equation (6) such that the variable and the parameter in the EDR model can be adapted to any scale of network topologies other than the cortical inter-areal connectivity:

(6) $$p\left(d_{n}\right)=\exp(-d_{n}/\alpha ),$$

where $d_{n}$ denotes the relative distance between two modules. That is, $d_{n}=d/d_{max}$, where the actual Euclidian distance *d* is divided by the largest distance of all the pairs of modules $d_{max}$. Regarding the control parameter, λ is replaced in Equation (1) by a new parameter $\alpha ={\left(\lambda d_{max}\right)}^{-1}$. The normalization constant *c* used in Equation (1) is eliminated because a predetermined number of inter-modular links are generated. Thus, $p(d_{n})$ can be regarded as a probability function that generates inter-modular links between any two given modules.

When generating the virtual layer, the following process is repeated until the predefined number $L_{inter}$ of inter-modular links are generated: (i) randomly choose a pair of modules; and (ii) probabilistically generate an inter-modular link according to $p(d_{n})$. In our proposed method, more than one inter-modular link can be assigned to a pair of modules because of the presence of multiple nodes in each module. Thus, after the deployment of inter-modular links, the endpoints of inter-modular links are assigned to nodes in the modules.

**Figure 3:**
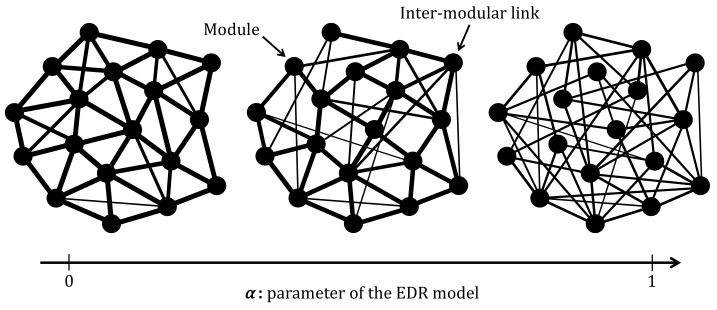
Relationship between parameter α and the topological shape.

Figure 3 shows the topologies generated using the procedure mentioned above. The circles represent modules, and the lines indicate the inter-modular links. The width of an inter-modular link corresponds to the number of links that exist between pairs of modules. As shown in Figure 3, when α is small, the inter-modular links are preferentially assigned to pairs of modules in close proximity. As α increases, the limitation on generating shorter inter-modular links is softened, and randomness on the connectivity increases.

### 3.3. Assigning Endpoints of Inter-Modular Links

As described in the previous subsection, inter-modular links are deployed among modules on the virtual layer. This subsection discusses the procedure to assign endpoints of inter-modular links to nodes from the viewpoint of node assortativity. That is, the endpoint nodes are chosen such that a specified value of assortativity ρ in Equation (5) is achieved on inter-modular connectivity for each pair of modules. By using this assignment scheme, the pairs of endpoints on which inter-modular links already exist are excluded. To obtain a suitable inter-modular connectivity that achieves a specified value of assortativity, the inter-modular links are repeatedly rewired. This process is performed stochastically by using the following procedure:

1. Two modules are randomly connected via a predetermined number of inter-modular links when constructing the virtual layer. Note that a pair of endpoint nodes does not have multiple edges.
2. The assortativity between the modules ρ is calculated. If ρ corresponds to the target value, then the set of connections at this point is retained. Otherwise, the following steps are executed.
3. An existing link between the modules, whose assortativity is farthest away from the target value, is deleted. If the current assortativity ρ is higher than the target value, the most assortative inter-modular link is selected, and vice versa.
4. A new link is created on two nodes in two different modules. The pair of nodes are randomly selected under the condition that the new link can move the assortativity ρ closer to the target value. Then, go back to Step 2.

**Figure 4:**
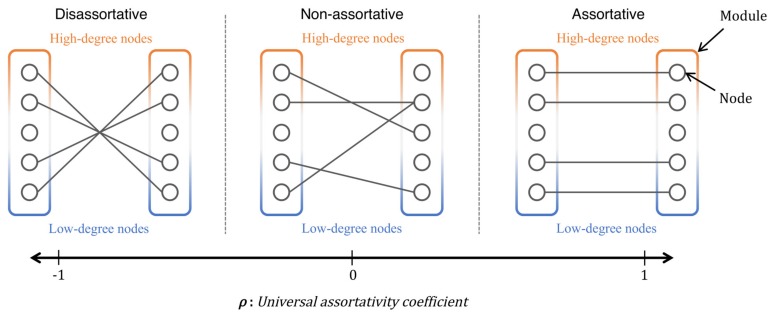
Relationship between universal assortativity coefficient and inter-modular connectivity.

The typical patterns of inter-modular connectivity corresponding to different values of the universal assortativity coefficient ρ are shown in Figure 4. When ρ>0, the two nodes with similar node degrees are chosen as endpoints of links; thus, a pair of high-degree nodes or low-degree nodes is connected. It should be noted that, even if a pair of nodes has a similar node degree, the two nodes with the average node degree are not preferred because they do not wield significant influence, as indicated by Equation (5). On the other hand, when ρ<0, a pair of two nodes with dissimilar degrees is assigned an inter-modular link. As ρ approaches to zero, the endpoint nodes for inter-modular links are more randomly chosen.

**Figure 5:**
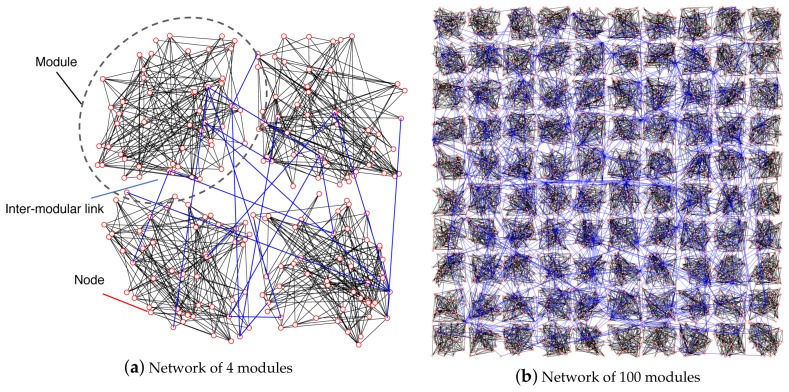
Examples of interconnected networks.

Figure 5 shows two example networks generated from the procedure above, where each link is unweighted and undirected.

## 4. Simulation Results

Extant research has studied the architecture for VWSN [18,19,20,21,22], but concrete strategies have not been investigated so far for constructing network topologies of the VWSN that satisfy various demands requested by the service providers. Since an enormous number of IoT devices and countless types of application services are deployed over the VWSN system, we consider that constructing the topologies focusing on basic topological nature is more essential than assuming a certain application service or traffic type. Therefore, our proposed method focuses on two points regarding topology construction for VWSN: assigning inter-modular links among modules based on the EDR model, and assigning endpoints of those inter-modular links on nodes based on network assortativity.

In this section, we present the results from computer simulations and discuss the performance of the VWSN topologies constructed using the proposed method in comparison with other conceivable network models. First, multiple networks are generated and connected with each other, thereby generating a VWSN topology. Then, several types of simulations are performed on the VWSN topologies, and their performance is measured in terms of communication efficiency, robustness, and network construction cost. The performance of the proposed method is evaluated relative to the performance of other comparative models.

### 4.1. Simulation Environment

First, we describe the computer simulation environment. This subsection describes the network models other than the EDR model that are used to assign links within and between modules. In addition, we also define the metrics to evaluate communication efficiency, robustness, and network construction cost. We also explain specific parameter settings for constructing the VWSN topologies for the computer simulations.

#### 4.1.1. Metrics

In this work, we study the influence of connectivity among modules on the performance of the system in terms of communication efficiency, robustness, and wiring cost. The metrics used for the evaluation of the performance in computer simulations are described as follows.

##### Communication Efficiency

To evaluate the communication efficiency of the VWSN topologies in terms of information transmission, we perform a packet routing simulation. We measure the time required for a data packet to pass from a source node to a destination node. In the realistic scenario for the IoT that is realized by the edge computing techniques, data collected and analyzed by the physical resources are passed to the edge servers. Then, the data are not uploaded to the cloud or the Internet side but the edge servers process by themselves in the edge computing scenario. When the data are proceeded and transmitted over the edge servers to the objective IoT module, they are delivered to the destination node. In the simulation, the packet routing is initiated at an arbitrary node, and the destination is also set on another arbitrary node. The packet is delivered according to the optimized path that minimizes the delay explained in the next paragraph. When a node receives a packet, the packet is forwarded to one of its neighbors that is included in the optimized path. The routing process is terminated when the data packet arrives at the destination node.

We conduct two types of packet routing simulation to measure the *service delay* and the *propagation delay*, respectively. At each instance that a packet arrives at a node, a service delay occurs according to an exponential distribution with service rate $\mu =1/D\;s^{-1}$. Furthermore, we assume that the propagation delay is Ds per 100m over the inter-modular links. These two types of delay are defined such that, on average, the service delay on a node is equal to the propagation delay over a 100m link. We assume *D* to be an arbitrary value because the value does not affect the simulation results in this study. The packet routing simulation, which focuses on the service delay, indicates how the hop count between two arbitrary nodes changes when the inter-modular connectivity is configured. On the other hand, the propagation delay indicates the change in the average route length in a given network topology.

##### Robustness

Robustness was evaluated by using algebraic connectivity [50]. Algebraic connectivity is a numeric value determined for a network topology from a graph-theoretical viewpoint. Fiedler et al. defined algebraic connectivity as the second smallest eigenvalue of the Laplacian matrix that is obtained from a network topology. It is well known that algebraic connectivity corresponds to the lower bound of both node connectivity and edge connectivity. The increase in algebraic connectivity leads to high robustness against node and link failures in the network topology because of the existence of multiple disjoint paths. In other words, a topology with high algebraic connectivity remains connected even if many nodes or links are removed [51,52,53,54].

Although there exist some other possible methods for evaluating the robustness of a network topology, in this study, we used algebraic connectivity because of the following advantages: (i) it does not depend on any parameter; (ii) it can uniquely specify robustness from just the shape of the network topology; and (iii) it can be calculated easily. Although the packet routing simulations for communication efficiency used in this study assume that bandwidth and network resources are sufficiently allocated and we do not consider network congestion, the metrics for robustness based on algebraic connectivity can indicate the degree of congestion in connection with the communication efficiency. When a generated VWSN topology shows high algebraic connectivity, the traffic can be distributed over many disjoint paths, and vice versa.

##### Wiring Cost

We define the wiring cost to assume the required cost for deploying the wireless inter-modular links among the gateways on the virtual layer. Given that we focus on wireless networks and cost arises from geometrical constraints in this study, the wiring cost is calculated as the sum of squares of the lengths of all inter-modular links based on the Friis transmission equation [55]. This equation predicts that the energy consumption for a wireless signal transmission increases with the square of the distance between transmitter and receiver. We exclude links within modules in the simulations because we do not change the intra-modular connectivity when evaluating the performance of the VWSN topologies.

#### 4.1.2. Network Models for Connectivity within Modules

Here, we describe the network models used to configure the connectivity within modules and between modules, respectively. First, we configure the connectivity patterns within modules. The effect of physical distance is considered to be negligible because the evaluation area for each module is considered to be sufficiently small. In this study, we focus on two types of common network models to determine the connectivity inside a module: the Barabási–Albert (BA) model [56] and the Erdös–Rényi (ER) model [57]. The BA model generates topologies whose degree distribution follows power-law, and, likewise, the ER model for Gaussian distribution. Both of the degree distributions are commonly observed in information networks.

##### Erdös–Rényi (ER) Model

The ER model belongs to a class of random network models [57]. The degree distribution of the ER model follows a Gaussian distribution that is similar to the distribution observed in WSNs [58,59,60]. For the construction of networks based on the ER model, we randomly choose a pair of nodes and connect them until the total number of intra-modular links is $L_{intra}$.

##### Barabáshi–Albert (BA) Model

The second type of networks corresponds to the BA model [56], which has been studied extensively as a class of complex networks. The BA model follows a power-law degree distribution and is characterized by the existence of extremely high-degree nodes (i.e., hub nodes) and a core cluster comprising hub nodes (i.e., rich-club). These characteristics are often observed in the real-world networks, such as airline networks, social networks, and the Internet. Thus, the BA model is commonly used in the field of information networking to generate Internet-like topologies.

For the topology construction, we first select a small set of nodes to generate an initial full-mesh topology, which is referred to as a seed. Then, we repeatedly add nodes to the seed. After the addition of a new node, *m* nodes of the existing topology are probabilistically chosen and connected to the new node via a link. The probability that the node *i* is chosen from the existing topology is given by $p_{i}=k_{i}/\Sigma_{j}k_{j}$, where $k_{i}$ denotes the degree of node *i* and $\Sigma_{j}k_{j}$ denotes the total degree of the existing topology; *m* is chosen such that almost the same number of intra-modular links ($L_{intra}$) are generated.

#### 4.1.3. Network Models for Connectivity between Modules

After generating the connections in modules, the network models for generating links between modules are described. In addition to the proposed EDR model, the short-link model and the long-link model are described, which consider the physical distance between modules. We also prepare the ER model as a null model. In contrast with the connectivity within modules, wherein the physical distance is not considered, the short-link and long-link models are used to compare the performances from the viewpoint of physical distance. Another point of difference from the intra-modular connectivity is that any pair of modules can have more than one link, whereas a pair of nodes inside the modules can have at most one link. This is because, even when a pair of modules has multiple links, each link can be assigned to different nodes inside both of the modules.

It should be noted that the following models can determine the pair of modules having inter-modular links; however, they do not consider the nodes in modules that behave as the endpoints of these inter-modular links. Hence, we also use assortativity for determining the endpoints of the inter-modular links, as described in Section 3.3.

##### Exponential Distance Rule (EDR) Model

Following the probability function $p(d_{n})$ in Equation (6), a pair of modules is repeatedly chosen and an inter-modular link is generated between this pair. The procedure is terminated when the total number of inter-modular links reaches $L_{inter}$.

##### Erdös–Rényi (ER) Model

We use the ER model to assign inter-modular links among modules to study the difference between our proposed model and a random model; the approach used is similar to the generation of links within modules. It should be noted that a pair of modules can have more than one link.

##### Short-Link (SL) Model

A network topology based on the SL model is composed of only links shorter than a threshold $R_{short}$ (in meters). We define this model for comparison with the EDR model and observe the difference in the performance if an inter-connected network does not contain long links. When constructing a topology, we randomly choose a pair of modules, generate a link if the distance is shorter than $R_{short}$, and repeat this process until $L_{inter}$ links have been generated.

##### Long-Link (LL) model

We define the LL model similar to the SL model by creating a topology with links longer than a threshold $R_{long}$.

#### 4.1.4. Parameter Settings

Table 1 shows the list of parameters configured during the construction of VWSN topologies. Each row contains a variable, description, and values for each parameter.

In the computer simulations, we assume that VWSN topologies are constructed on an $E\times E\;m^{2}$ square area and *N* nodes are deployed as physical nodes. The area is divided into *M* smaller regions in a grid pattern, and each area contains an equal number of $N^{\prime }=N/M$ nodes. The physical nodes in an area are inter connected via $L_{intra}$ links, and form a module on the physical layer. The value for $L_{intra}$ is assumed such that a node corresponds to three intra-modular links. Then, the *M* modules are connected by $L_{inter}$ inter-modular links to form the virtual layer. The details of the values of $L_{inter}$ are provided in the following sections. Regarding the connectivity between modules, the parameter α controls the inter-modular connectivity of a topology based on the EDR model, and the parameter ρ controls the inter-modular assortativity of a pair of modules. $R_{short}$ and $R_{long}$ limit the length of links when constructing VWSN topologies based on the SL model and the LL model, respectively.

The values for parameter settings differ for each type of simulation, from Section 4.2 to Section 4.4. Thus, the detailed values are described for each evaluation.

### 4.2. Basic Property of Assortativity on 4-Module Networks

First, we conduct computer simulations using small-scale VWSN topologies. This subsection focuses on the assortativity of intra-modular connectivity and evaluates its influence on communication efficiency and robustness. In other words, before considering which pair of modules should have inter-modular links, we determine the nodes within the modules that should be assigned as endpoints of inter-modular links. Therefore, we do not consider the physical distance when evaluating the performance, and neither do we apply network models for connectivity between modules, as described in Section 4.1.3.

The parameter settings are shown in Table 1. A VWSN topology is composed of M=4 modules, as shown in Figure 5. Each pair of modules is equally assigned five inter-modular links, and a VWSN topology has $L_{inter}=5\times 6=30$ inter-modular links given that there are ${}_{4}C_{2}=6$ possible combinations for connecting two out of four modules. The possible range of inter-modular assortativity for a VWSN topology is determined by the network models used for connectivity within the modules. Assortativity in the ER model varied from -0.05 to 0.05, whereas, in the BA model, it varied from -0.04 to 0.1. This difference is indicative of the fact that the distribution of node degree was more strongly biased in the BA model. In other words, the number of high-degree nodes was lower in the BA model, and, thus, such nodes were rarely connected to other nodes of the same degree, thereby decreasing assortativity.

#### 4.2.1. Robustness

**Figure 6:**
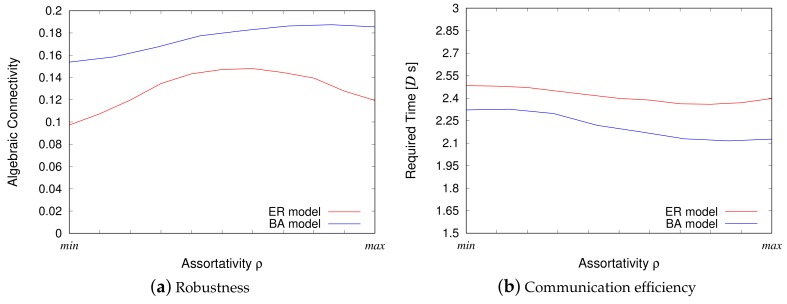
Simulation results for 4-module networks. (min, max) corresponds to (-0.65, 0.05) and (-0.04, 0.10) for the ER and BA models, respectively.

Figure 6a shows the algebraic connectivity for the ER model and the BA model. The *y*-axis represents algebraic connectivity, and the *x*-axis represents intra-modular assortativity. Regarding the *x*-axis, we unite the different assortativity ranges of the ER and BA models to compare the performance more clearly. Each figure is the compilation of the results from 100 computer simulations, thus enabling the generation of a VWSN topology and measurement of the algebraic connectivity. As explained in the section above, high algebraic connectivity indicates that the network topology is robust against node and link failures.

Regarding the ER model, VWSN topologies with non-assortative or slightly assortative inter-modular connectivity exhibit higher robustness. It can also be said that assortative connectivity is a better indicator of high robustness than disassortative connectivity. These characteristics are closely associated with the degree of endpoint nodes of inter-modular links. When a topology is assortative, the nodes of similar degree are connected with each other. On the other hand, in a disassortative topology, the nodes tend to be connected if the degree is dissimilar. Therefore, all inter-modular links of a VWSN topology of disassortative connectivity have a high-degree node on one side, and a low-degree node on the other side. A low-degree node is located on the periphery of its module, and all the inter-modular links of a disassortative topology do not contribute to the creation of disjoint paths among nodes. This characteristic can explain why a disassortative topology exhibits the lowest robustness. Regarding an assortative topology, one half of the inter-modular links connect two high-degree nodes, and the other half connect two low-degree nodes. In contrast, in the non-assortative topology, endpoints for inter-modular links are randomly chosen. From the result in Figure 6a, we can conclude that inter-modular links of two low-degree nodes in an assortative topology offset the benefit of links between high-degree nodes, and random connectivity between modules is preferred in terms of generating much more disjoint paths in a VWSN topology. Therefore, non-assortative topologies exhibit the highest robustness, followed by assortative topologies and disassortative topologies, in that order.

On the other hand, for the BA model, high assortative inter-modular connectivity of a VWSN topology is directly associated to a higher level of robustness. This interesting characteristic may be attributed to the small fraction of nodes contributing to high-degree nodes in the BA model owing to the power-law distribution. Assortativity is calculated based on the difference between the degree of each node to the average node degree. To achieve high assortativity, in the BA model, connection between two nodes with high degrees is preferred because node degree gap between high-degree nodes and the average-degree nodes is much greater than the gap between low-degree nodes and the average-degree nodes. Therefore, in an assortative VWSN topology of the BA model, almost all the inter-modular links contain high-degree nodes at both the endpoints and create many disjoint paths among nodes. On the other hand, for the ER model, only half of them are between two high-degree nodes. This results in the high robustness of an assortative VWSN topology based on the BA model.

When comparing results from the ER model and the BA model, the latter shows more robust features for any given value of assortativity. This can be attributed to the difference in node degree distribution. In a topology based on the BA model, the dense central core of hub nodes, i.e., rich club, tightly connects all the nodes and strengthens the connectivity. This decreases the diameter and shrinks the topological shape of the network. Thus, a few failures do not split the topology of the BA model. In contrast, a topology based on the ER model is more sparsely and uniformly connected, and can be broken easily into smaller clusters. These characteristics contribute to the difference in the algebraic connectivity between the ER and BA models.

#### 4.2.2. Communication Efficiency

Figure 6b shows the results obtained from the packet routing simulations for the ER and BA models. The *y*-axis represents the average time required for the data packet to arrive at a destination node from another source node in the VWSN topology. Although in Section 4.1.1 we conducted two types of packet routing simulations that consider propagation delay on links and service delay on nodes, this section provides only the results of simulations that consider service delay. This is because physical distance is assumed negligible for the 4-module VWSN networks. We generated 100 topologies for each model and 1000 packets on each topology to measure the communication delay.

The two results from the ER model and the BA model indicate a similar tendency in terms of information transmission speed. When inter-modular links of a VWSN topology are disassortatively connected, the transmission speed, i.e., communication efficiency, is the lowest. As the assortativity for inter-modular connectivity increases, the transmission speed also increases. This is because, for large values of assortativity, connections will be composed of two high-degree nodes as endpoints. Therefore, it is much easier for information to diffuse over the entire topology by passing through the connections of the two influential nodes. However, when the assortativity is maximized, the transmission speed decreases slightly. This reduction in transmission speed can be attributed to the fact that nodes with the highest or lowest degrees have to be connected to multiple inter-modular links at the same time so that assortativity is maximized; this results in inefficient spread of information over the network topology.

Moreover, it was observed that VWSN topologies based on the BA model diffuse information at a faster rate than those of the ER model. This can be explained by the fact that connecting the hub nodes in different modules based on the BA model enables faster transmission of information.

### 4.3. Evaluation of the Proposed Model

In the previous evaluation, VWSN topologies composed of four modules were used in order to focus on the assortativity between modules and its effect on the performance of communication efficiency and robustness. In this subsection, we also take geometric constraints and the lengths of inter-modular links into account. Therefore, we expand the scale of the topology into M=100 modules and apply the network models explained in Section 4.1.3 for connectivity between modules.

As shown in Table 1, equal values are used for $N^{\prime }$ and $L_{intra}$ because the VWSN topology has been shifted into a larger scale in the square region. The number of inter-modular links $L_{inter}$ is determined to be $L_{inter}=3\times M=300$ such that three inter-modular links are added per module. Because the detailed effect of assortativity ρ on the performance has been already investigated in the previous section, hereafter, we focus on just three values of ρ. $\rho_{min}$ corresponds to the minimum ρ that leads to the most disassortative connectivity on a given VWSN topology, and, similarly, $\rho_{max}$ corresponds to the maximum ρ. $\rho_{zero}$ indicates that ρ=0 and that the topology has non-assortative connectivity between modules. We vary the parameter α for the EDR model in the range of [0.025,0.8] in order to configure the pattern of inter-modular connectivity. We affix the lower limit to 0.25 because of the difficulty in generation of a connected topology with smaller α; in addition, we affix the upper limit to 0.08 because we confirmed that the topology shape does not change significantly even if we use larger α; the topology becomes similar to that of the ER model. Each result is obtained from 100 repetitions of computer simulations for each pattern of a given VWSN topology.

#### 4.3.1. Robustness

**Figure 7:**
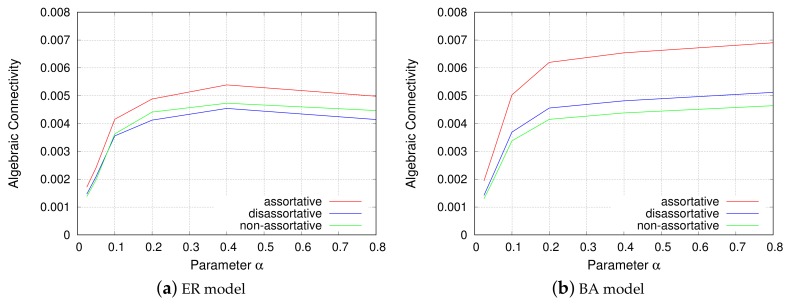
Robustness.

Figure 7 shows the relationship between algebraic connectivity, the parameter α from the EDR model, and the inter-modular assortativity. The *y*-axis represents algebraic connectivity, while the *x*-axis represents the parameter α. The two subfigures contain three curves, each corresponding to assortative, disassortative, and non-assortative connectivity between modules, respectively.

For every curve in both the subfigures for the ER and BA models, it can be said that a VWSN topology cannot achieve high robustness with a small parameter α. The smaller the parameter α is, the shorter the links that the VWSN topology has; that is, no long links exist that connect distant modules. Therefore, the modules are locally connected with each other and form several clusters. The topology is fragile and can be easily broken into clusters when node or link failure occurs. Increase in parameter α is characterized by the appearance of long links, and the connectivity between multiple modules is rendered more complicated, as in the case of a random graph (the ER model). Hence, the number of disjoint paths increases and the VWSN topology becomes redundant.

Regarding the assortativity, it should be noted that an assortative topology exhibits the highest algebraic connectivity, i.e., the most robust feature, in both the ER and BA models. In the ER model of the 4-module networks, however, we confirmed that non-assortative or slightly assortative connectivity achieves the highest robustness. This may be attributed to the difference in the number of modules; in a 4-module network, each module was connected to all the other modules by one hop. However, in this case, a VWSN topology is composed of 100 modules, and, therefore, most pairs of modules are indirectly connected. From this result, it can be conjectured that, when a path between two nodes passes through multiple modules, the inter-modular links composed of two high-degree nodes contribute to the increase in the number of disjoint paths. This results in the increase of algebraic connectivity, i.e., robustness. The effect of connections between high-degree nodes is larger in the BA model, and an assortative topology achieves significantly higher robustness.

#### 4.3.2. Communication Efficiency

**Figure 8:**
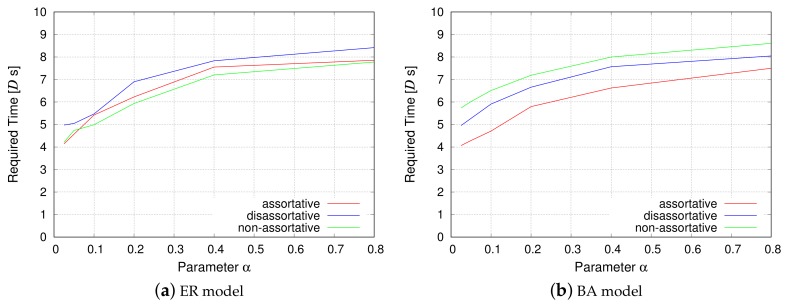
Communication efficiency (propagation delay).

**Figure 9:**
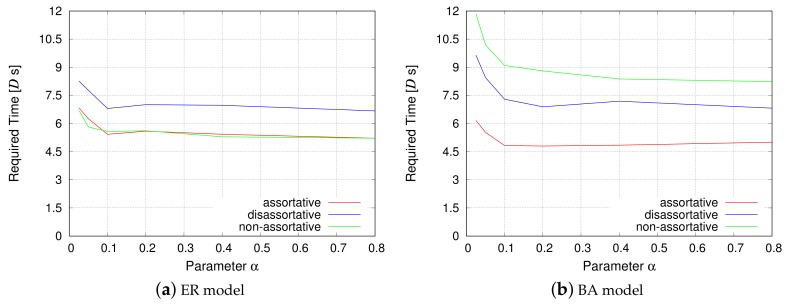
Communication efficiency (service delay).

Although the physical distance and the propagation delay are not considered in the evaluation for 4-module networks, the packet routing simulations described in this section consider both the service delay and propagation delay. Figure 8 corresponds to the simulation that measures the propagation delay, and Figure 9 corresponds to the simulation that measures the service delay. The *y*-axis represents the average time required for the data packet to pass from a source node to a destination node in the VWSN topology, and the *x*-axis represents the parameter α from the EDR model.

Figure 8 shows that, as the parameter α decreases, the propagation delay is reduced. When α is small, the EDR model tends to generate shorter inter-modular links. As a result, the connectivity among modules approaches that of a grid topology. Therefore, any given pair of modules will be roughly connected in a straight line. This enables a VWSN topology with small α to decrease the propagation delay. On the other hand, when α is large, the number of long connections between modules increases. Hence, the shortest paths among modules follow a zigzag pattern, as opposed to a straight line. Such zigzag paths lead to an increase in the propagation delay. In contrast, the required time for information transmission increases for small α when considering service delay, as shown in Figure 9. Even if the shortest path for a pair of modules is along a straight line, the path is composed of many short inter-modular links. Since the service delay occurs on every hop, the time required for information transmission is large when α is small.

As for the inter-modular assortativity, in Section 4.2, we showed that assortative inter-modular connectivity can minimize the service delay for a 4-module network. In that sense, we can confirm a similar tendency in Figure 8 and Figure 9, which indicates that assortative topology exhibits the best performance. However, the performance of a non-assortative topology is different for the ER model and the BA model. This may be attributed to the difference of node degree distribution. As mentioned earlier, half of the inter-modular links in an assortative topology are composed of low-degree nodes in the ER model. They offset the benefit of the rest of the inter-modular links composed of high-degree nodes. For the ER model, the results indicate that the performance of random connectivity in a non-assortative topology is almost similar to that of a mixture of high-degree connections and low-degree connections in an assortative topology. Meanwhile, the non-assortative topology exhibits the slowest rate of information transmission for the BA model, even though a non-assortative topology is slightly better than the disassortative topology for 4-module networks, as shown in Figure 6b. This is because, when information passes through multiple modules, hub nodes, i.e., extremely high-degree nodes, have greater influence for the BA model, and inter-modular links of a non-assortative topology do not contain hub nodes.

#### 4.3.3. Wiring Cost

**Figure 10:**
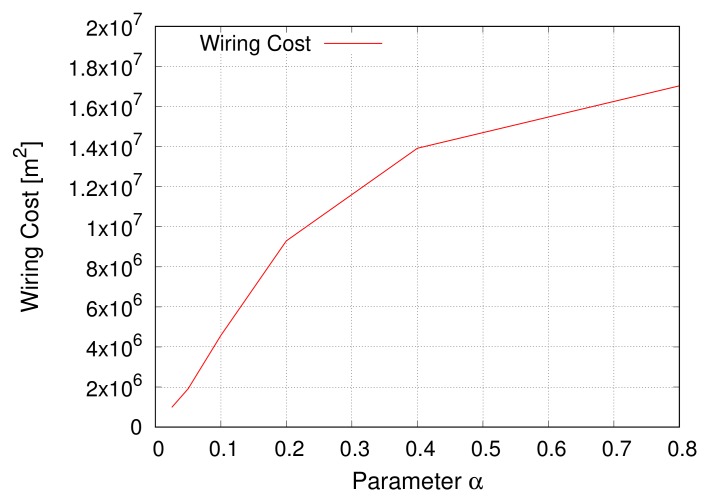
Wiring cost.

In Figure 10, the *y*-axis represents the sum of squares of the lengths of all inter-modular links, as wiring cost is based on the *Friis transmission equation* [55]. As explained in Section 4.1.1, we do not consider the cost of links within modules. The *x*-axis corresponds to the parameter α of the EDR model. In Figure 10, assortativity or network models for intra-modular connectivity are assumed to have no effect on the evaluation, and hence are omitted.

The result shows a monotonous change in the wiring cost when the parameter α is varied. For small values of α, the EDR model probabilistically tends to generate shorter links according to Equation (6). As the parameter α increases, the restriction on generating long links is softened.

### 4.4. Comparison with Other Network Models

We investigate the effect on the performances of the difference in network models for inter-modular connectivity. We choose 0.025, 0.1, and 0.4 as representative values for the parameter α of the EDR model to construct a VWSN topology. In addition, we prepare the ER model, SL model, and LL model, which are explained in Section 4.1.3, and compare the performance of these models. As shown in Table 1, only the parameters of α, $R_{short}$, and $R_{long}$ are changed from those in Section 4.3. Each result is obtained from 100 repetitions of computer simulations for each pattern of a given VWSN topology.

#### 4.4.1. Robustness

**Figure 11:**
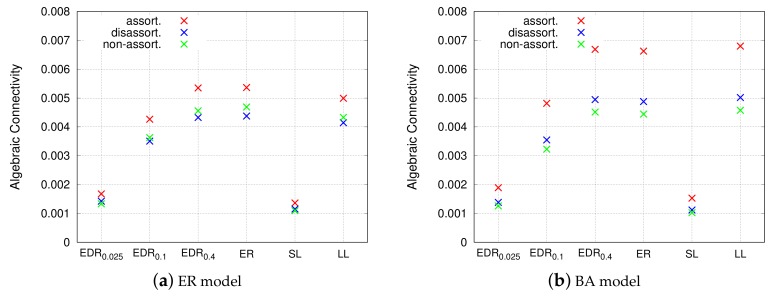
Robustness

Figure 11 shows the algebraic connectivity for each network model. The order of the algebraic connectivity of the network models on the *x*-axis is similar for the ER and BA models. The ${\mathrm{EDR}}_{0.025}$ and the SL model equally mark the lowest values for the algebraic connectivity. The SL model generates no long links, and the ${\mathrm{EDR}}_{0.025}$ also does not generate long links due to the limitation imposed by Equation (6). The resultant grid-like topologies are easily broken into small clusters in the event of failures, as mentioned in Section 4.3. On the other hand, the ${\mathrm{EDR}}_{0.4}$, ER, and LL models equally exhibit the highest algebraic connectivity. This result is interesting because the LL model generates only long links and no short links, unlike the other two models. From this viewpoint, it can be said that long inter-modular links are more important for the creation of disjoint paths and to improve redundancy of a VWSN topology. The assortative topology has the highest robustness, as explained in Section 4.3.

#### 4.4.2. Communication Efficiency

**Figure 12:**
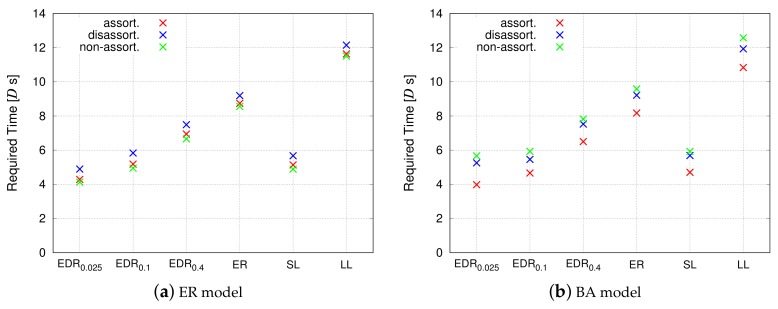
Communication efficiency (propagation delay).

**Figure 13:**
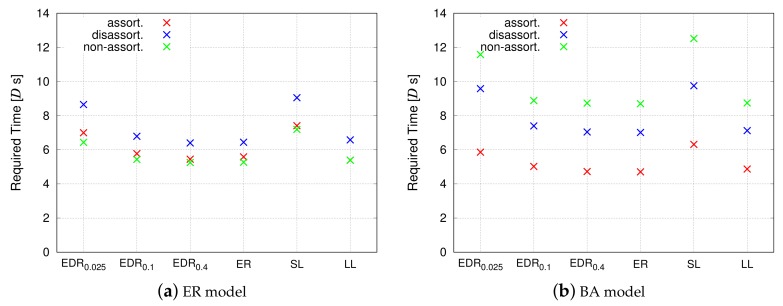
Communication efficiency (service delay).

Figure 12 and Figure 13 show the time required for packet routing, and focus on the propagation delay and service delay, respectively. Regarding propagation delay, the ${\mathrm{EDR}}_{0.025}$ model exhibits the smallest delay, followed by the ${\mathrm{EDR}}_{0.1}$ and SL models. For shorter inter-modular links, the VWSN topology approaches a grid-like topology; the route between two nodes is along a straight line. On the other hand, the LL model shows a considerably slow diffusion speed. In a topology based on the LL model, there are no short links, and the data is required to undertake a detour when it passes around the topology. Regarding service delay, the SL model exhibits the poorest performance, followed by the ${\mathrm{EDR}}_{0.0.025}$ model. All the other models equally show the smallest delay.

Notable characteristics are observed on the ${\mathrm{EDR}}_{0.1}$, which achieves the best performance in terms of propagation delay and service delay. The procedure for generating links in the EDR model is probabilistic. Hence, the topologies of ${\mathrm{EDR}}_{0.1}$ can contain a small number of long inter-modular links, though almost all the links are short. From Figure 12 and Figure 13, it can be confirmed that many short links in the ${\mathrm{EDR}}_{0.1}$ can help achieve a propagation similar to that of the SL model, and only a small number of long links are required to achieve a small service delay as that of the LL model.

#### 4.4.3. Wiring Cost

**Figure 14:**
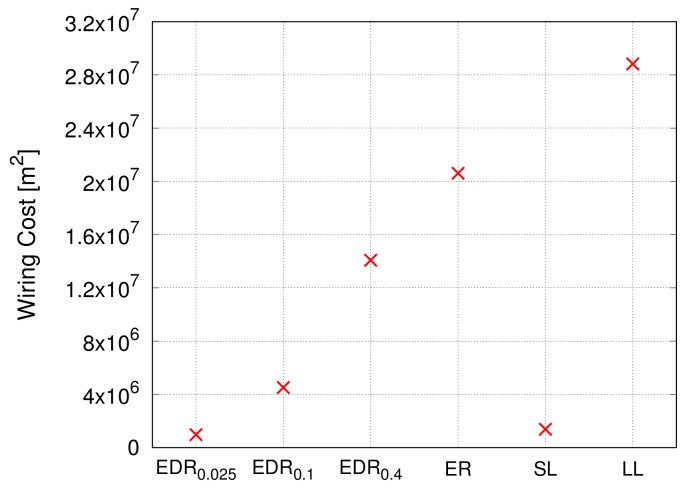
Wiring cost.

The performance for wiring cost shown in Figure 14 is similar to that of communication efficiency of the propagation delay in Figure 12. It can be said that the ratio of short inter-modular links is closely linked to both results. The ${\mathrm{EDR}}_{0.025}$ and the SL model display the lowest cost, whereas the LL model exhibits an extremely high cost. The results simply reflect the procedure of generating inter-modular links: ${\mathrm{EDR}}_{0.025}$ and the SL models generate short links, and the LL model generates long links. We can confirm that the cost of ${\mathrm{EDR}}_{0.1}$ is also much smaller than the other models except for ${\mathrm{EDR}}_{0.025}$ and SL models. This implies that, although the performance of the ${\mathrm{EDR}}_{0.1}$ and ER models shown in Figure 11 and Figure 13 is similar, ${\mathrm{EDR}}_{0.1}$ is still biased towards generating shorter links when compared with the ER model, which generates links of the average length. If α is further increased, the inter-modular connectivity approaches that of the ER model.

## 5. Conclusions

In this study, we proposed and evaluated a method to construct a VWSN topology. Since there are an enormous number of IoT devices and countless types of application services on the future VWSN, in order to satisfy the required performances such as communication efficiency, robustness, and construction cost, we focused on two basic topological properties: *which pair of modules should be assigned inter-modular links*, and *which nodes in the modules should be assigned endpoints of the inter-modular links*. For the former, we focused on an inter-modular connectivity model based on the cerebral cortex of a mammalian brain, which is referred to as the EDR model. For the latter, we focused on assortativity, which is an important property that characterizes the modular connectivity structure of human brain networks.

The proposed brain-inspired method constructs the virtual layer based on the EDR model, i.e., inter-modular links are assigned among a set of modules. The proposed method exhibited a trade-off between the metrics used in the computer simulations. When the parameter α shifted towards zero, service delay increased and robustness decreased, while propagation delay and wiring cost decreased. By contrast, if α is increased, the performance is good in terms of service delay and robustness, whereas propagation delay and wiring costs tend to deteriorate.

In comparison with other network models, we also confirmed that the proposed model can simultaneously achieve high performance in terms of robustness, communication efficiency, and construction cost when the parameter α is set around 0.1 in the above-mentioned trade-off. We revealed that robustness could be enhanced while suppressing service delay, when a small number of long inter-modular links are generated in a VWSN topology. This leads to a reduction in the construction cost, and propagation delay is also reduced by the existence of dozens of short inter-modular links in the topology. When α is approximately 0.1, most inter-modular links generated in the construction process are short, but a few long links are also probabilistically generated at the same time. These long links enable our proposed model to achieve high robustness and low service delay. Correspondingly, when an inter-modular topology of the cerebral cortex is reproduced, Markov et al. used a similar parameter setting of: $\alpha ={\left(\lambda d_{max}\right)}^{-1}={\left(0.180\times 58.2\right)}^{-1}≃0.0954$ [61]. This implies that brain networks also deal with this trade-off and realize high performance. In that sense, we also showed that the characteristics of brain networks are applicable to the VWSN topology.

With respect to inter-modular assortativity, topologies with high asssortativity revealed high performance with regard to both robustness and communication efficiency. We can also confirm that there exists a difference when the ER and BA models are used for connectivity within modules. When using the BA model, most inter-modular links consist of hub nodes, which have greater topological importance. This characteristic of the BA model enables the links to contribute the performance more than the ER model. We also confirmed that an assortative topology becomes superior when the number of modules is increased. This is because inter-modular links of high-degree nodes exert a greater influence when a pair of nodes is connected through many more indirect routes. From these observations, we can establish that connecting two high-degree nodes to generate inter-modular links contributes to performance enhancement in terms of robustness and communication efficiency. This is particularly applicable to the case when each module has scale-free-like connectivity and the number of modules is significantly large.

In real world IoT networks, various constraints in the environment or service demands from the providers may affect the construction of a VWSN topology over edge computing systems and WSNs. For instance, an enormous number of IoT devices are assumed to consist of a VWSN topology as a physical layer in this study. In such a situation, it is essential to take into consideration the battery life of the devices and communication distances. Meanwhile, if the application service that runs over the VWSN topology is a life-critical system, high robustness against computer-virus infections or network failures are of critical importance. Regarding the assignment of inter-modular links among modules, the infrastructure providers can construct a VWSN topology that is suited to specific situations by using our proposed model and setting the parameter α around 0.1. With respect to choosing endpoint nodes for the inter-modular links, it can be summarized that assortatively inter-connecting high-degree nodes enhances both robustness and communication efficiency. Through the discussion in this paper, we conclude that our proposed methods can help design VWSN architectures that can deal with various demands that may arise in actual IoT scenarios.

## Figures and Tables

**Table 1:** Parameter description.

Variable	Description	Values for Section 4.2	Values for Section 4.3	Values for Section 4.4
*E*	Length of the side of evaluation area	100m	500m	500m
*N*	Number of nodes	200	5000	5000
*M*	Number of modules	4	100	100
$N^{\prime }$	Number of nodes in each module	50	50	50
$L_{intra}$	Number of intra-modular links	150	150	150
$L_{inter}$	Number of inter-modular links	30	300	300
ρ	Parameter of inter-modular assortativity (Eq. (5))	variable	$\rho_{min},\;\rho_{zero},\;\rho_{max}$	$\rho_{min},\;\rho_{zero},\;\rho_{max}$
α	Parameter of the EDR model (Eq. (6))	-	[0.025,0.8]	0.025,0.1,0.8
$R_{short}$	Upper limit of link length for the SL model	-	-	10m
$R_{long}$	Lower limit of link length for the LL model	-	-	20m

## Abbreviations

The following abbreviations are used in this manuscript:

WSN	Wireless Sensor Network
VSN	Virtualized Service Network
VWSN	Virtualized Wireless Service Network
EDR	Exponential Distance Rule
ER	Erdös Rényi
BA	Barabási Albert
SL	Short-Link
LL	Long-Link

## References

[1] Akyildiz I., Su W., Sankarasubramaniam Y., Cayirci E. (2002). Wireless sensor networks: A survey. Comput. Netw..

[2] Raza U., Kulkarni P., Sooriyabandara M. (2017). Low Power Wide Area Networks: An Overview. IEEE Commun. Surv. Tutor..

[3] Nolan K.E., Guibene W., Kelly M.Y. An evaluation of low power wide area network technologies for the Internet of Things. Proceedings of the 2016 International Wireless Communications and Mobile Computing Conference (IWCMC).

[4] Shi W., Cao J., Zhang Q., Li Y., Xu L. (2016). Edge Computing: Vision and Challenges. IEEE Internet Things J..

[5] Satyanarayanan M. (2017). The Emergence of Edge Computing. Computer.

[6] Yi S., Hao Z., Qin Z., Li Q. Fog Computing: Platform and Applications. Proceedings of the 2015 Third IEEE Workshop on Hot Topics in Web Systems and Technologies (HotWeb).

[7] Dastjerdi A.V., Buyya R. (2016). Fog Computing: Helping the Internet of Things Realize Its Potential. Computer.

[8] Stankovic J.A. (2014). Research Directions for the Internet of Things. IEEE Internet Things J..

[9] Ochoa S.F., Fortino G., Fatta G.D. (2017). Cyber-physical systems, internet of things and big data. Future Gener. Comput. Syst..

[10] Xu L.D., He W., Li S. (2014). Internet of Things in Industries: A Survey. IEEE Trans. Ind. Inform..

[11] Whitmore A., Agarwal A., Da Xu L. (2015). The Internet of Things—A survey of topics and trends. Inf. Syst. Front..

[12] Han B., Gopalakrishnan V., Ji L., Lee S. (2015). Network function virtualization: Challenges and opportunities for innovations. IEEE Commun. Mag..

[13] Mijumbi R., Serrat J., Gorricho J.L., Bouten N., Turck F.D., Boutaba R. (2016). Network Function Virtualization: State-of-the-Art and Research Challenges. IEEE Commun. Surv. Tutor..

[14] Nunes B.A.A., Mendonca M., Nguyen X.N., Obraczka K., Turletti T. (2014). A Survey of Software-Defined Networking: Past, Present, and Future of Programmable Networks. IEEE Commun. Surv. Tutor..

[15] Kreutz D., Ramos F.M.V., Veríssimo P.E., Rothenberg C.E., Azodolmolky S., Uhlig S. (2015). Software-Defined Networking: A Comprehensive Survey. Proc. IEEE.

[16] Zhou X., Li R., Chen T., Zhang H. (2016). Network slicing as a service: Enabling enterprises’ own software-defined cellular networks. IEEE Commun. Mag..

[17] Zhang H., Liu N., Chu X., Long K., Aghvami A.H., Leung V.C.M. (2017). Network Slicing Based 5G and Future Mobile Networks: Mobility, Resource Management, and Challenges. IEEE Commun. Mag..

[18] Liang C., Yu F.R. (2015). Wireless network virtualization: A survey, some research issues and challenges. IEEE Commun. Surv. Tutor..

[19] Khan I., Belqasmi F., Glitho R., Crespi N., Morrow M., Polakos P. (2016). Wireless sensor network virtualization: A survey. IEEE Commun. Surv. Tutor..

[20] Richart M., Baliosian J., Serrat J., Gorricho J.L. (2016). Resource slicing in virtual wireless networks: A survey. IEEE Trans. Netw. Serv. Manag..

[21] Kobo H.I., Abu-Mahfouz A.M., Hancke G.P. (2017). A Survey on Software-Defined Wireless Sensor Networks: Challenges and Design Requirements. IEEE Access.

[22] Modieginyane K.M., Letswamotse B.B., Malekian R., Abu-Mahfouz A.M. (2018). Software defined wireless sensor networks application opportunities for efficient network management: A survey. Comput. Electr. Eng..

[23] Morabito R., Cozzolino V., Ding A.Y., Beijar N., Ott J. (2018). Consolidate IoT Edge Computing with Lightweight Virtualization. IEEE Netw..

[24] Baktir A.C., Ozgovde A., Ersoy C. (2017). How Can Edge Computing Benefit From Software-Defined Networking: A Survey, Use Cases, and Future Directions. IEEE Commun. Surv. Tutor..

[25] Morabito R., Beijar N. Enabling Data Processing at the Network Edge through Lightweight Virtualization Technologies. Proceedings of the 2016 IEEE International Conference on Sensing, Communication and Networking (SECON Workshops).

[26] Morabito R., Petrolo R., Loscrí V., Mitton N. Enabling a lightweight Edge Gateway-as-a-Service for the Internet of Things. Proceedings of the 2016 7th International Conference on the Network of the Future (NOF).

[27] Mayoral A., Vilalta R., Casellas R., Martinez R., Munoz R. (2016). Multi-tenant 5G Network Slicing Architecture with Dynamic Deployment of Virtualized Tenant Management and Orchestration (MANO) Instances. Proceedings of 42nd European Conference on Optical Communication (ECOC 2016).

[28] Kaiwartya O., Abdullah A.H., Cao Y., Lloret J., Kumar S., Shah R.R., Prasad M., Prakash S. (2017). Virtualization in Wireless Sensor Networks: Fault Tolerant Embedding for Internet of Things. IEEE Internet Things J..

[29] Morabito R. (2017). Virtualization on Internet of Things Edge Devices With Container Technologies: A Performance Evaluation. IEEE Access.

[30] Li J., Jin J., Yuan D., Zhang H. (2018). Virtual Fog: A Virtualization Enabled Fog Computing Framework for Internet of Things. IEEE Internet Things J..

[31] Roh W., Seol J.Y., Park J., Lee B., Lee J., Kim Y., Cho J., Cheun K., Aryanfar F. (2014). Millimeter-wave beamforming as an enabling technology for 5G cellular communications: theoretical feasibility and prototype results. IEEE Commun. Mag..

[32] Kutty S., Sen D. (2016). Beamforming for Millimeter Wave Communications: An Inclusive Survey. IEEE Commun. Surv. Tutor..

[33] Bullmore E., Sporns O. (2012). The economy of brain network organization. Nat. Rev. Neurosci..

[34] Sporns O., Betzel R.F. (2016). Modular brain networks. Ann. Rev. Psychol..

[35] Ercsey-Ravasz M., Markov N.T., Lamy C., Van Essen D.C., Knoblauch K., Toroczkai Z., Kennedy H. (2013). A predictive network model of cerebral cortical connectivity based on a distance rule. Neuron.

[36] Murakami M., Leibnitz K., Kominami D., Shimokawa T., Murata M. (2017). Constructing Virtual IoT Network Topologies with a Brain-inspired Connectivity Model. Proceedings of the 11th International Conference on Ubiquitous Information Management and Communication (IMCOM’17).

[37] Brandes U. (2008). On variants of shortest-path betweenness centrality and their generic computation. Soc. Netw..

[38] Klimm F., Bassett D.S., Carlson J.M., Mucha P.J. (2014). Resolving Structural Variability in Network Models and the Brain. PLoS Comput. Biol..

[39] Murakami M., Ishikura S., Kominami D., Shimokawa T., Murata M. (2017). Robustness and efficiency in interconnected networks with changes in network assortativity. Appl. Netw. Sci..

[40] Bassett D.S., Bullmore E. (2006). Small-world brain networks. Neuroscientist.

[41] Eguiluz V.M., Chialvo D.R., Cecchi G.A., Baliki M., Apkarian A.V. (2005). Scale-free brain functional networks. Phys. Rev. Lett..

[42] Van den Heuvel M.P., Stam C.J., Boersma M., Pol H.H. (2008). Small-world and scale-free organization of voxel-based resting-state functional connectivity in the human brain. Neuroimage.

[43] Sporns O. (2010). Networks of the Brain.

[44] Meunier D., Lambiotte R., Bullmore E.T. (2010). Modular and hierarchically modular organization of brain networks. Front. Neurosci..

[45] Meunier D., Lambiotte R., Fornito A., Ersche K., Bullmore E. (2009). Hierarchical modularity in human brain functional networks. Front. Neuroinform..

[46] Knoblauch K., Ercsey-Ravasz M., Kennedy H., Toroczkai Z. (2016). The brain in space. Micro-, Meso-and Macro-Connectomics of the Brain.

[47] Kaiser M., Hilgetag C.C. (2006). Nonoptimal component placement, but short processing paths, due to long-distance projections in neural systems. PLoS Comput. Biol..

[48] Zhang G.Q., Cheng S.Q., Zhang G.Q. (2012). A universal assortativity measure for network analysis. arXiv.

[49] Boccaletti S., Latora V., Moreno Y., Chavez M., Hwang D.U. (2006). Complex networks: Structure and dynamics. Phys. Rep..

[50] Fiedler M. (1973). Algebraic connectivity of graphs. Czechoslov. Math. J..

[51] Jamakovic A., Van Mieghem P. On the Robustness of Complex Networks by Using the Algebraic Connectivity. Proceedings of the International Conference on Research in Networking.

[52] Jamakovic A., Uhlig S. On the relationship between the algebraic connectivity and graph’s robustness to node and link failures. Proceedings of the 2007 Next Generation Internet Networks.

[53] Alenazi M.J., Çetinkaya E.K., Sterbenz J.P. (2014). Cost-efficient algebraic connectivity optimisation of backbone networks. Opt. Switch. Netw..

[54] Sydney A., Scoglio C., Gruenbacher D. (2013). Optimizing algebraic connectivity by edge rewiring. Appl. Math. Comput..

[55] Friis H.T. (1946). A note on a simple transmission formula. Proc. Inst. Radio Eng..

[56] Barabási A.L., Albert R. (1999). Emergence of scaling in random networks. Science.

[57] Erdös P., Rényi A. (1959). On random graphs, I. Publ. Math. (Debrecen).

[58] Kawahigashi H., Terashima Y., Miyauchi N., Nakakawaji T. Modeling ad hoc sensor networks using random graph theory. Proceedings of the Second IEEE Consumer Communications and Networking Conference, 2005, (CCNC 2005).

[59] Onat F.A., Stojmenovic I. Generating Random Graphs for Wireless Actuator Networks. Proceedings of the 2007 IEEE International Symposium on a World of Wireless, Mobile and Multimedia Networks.

[60] Ding L., Guan Z.H. (2008). Modeling wireless sensor networks using random graph theory. Phys. A Stat. Mech. Appl..

[61] Markov N.T., Ercsey-Ravasz M., Lamy C., Ribeiro Gomes A.R., Magrou L., Misery P., Giroud P., Barone P., Dehay C., Toroczkai Z. (2013). The role of long-range connections on the specificity of the macaque interareal cortical network. Proc. Natl. Acad. Sci. USA.

